# Effects of microbial-phytase co-fermentation of sorghum on growth performance, nutrient utilization, digestive enzyme activities and cecal microbiota in meat ducks

**DOI:** 10.1016/j.psj.2026.107526

**Published:** 2026-07-30

**Authors:** Junlei Chang, Yu Deng, Jianqi Yang, Yang Liu, Leizheng Zhang, Dongmei Xie, Xuemei Ding, Jianping Wang, Yan Liu, Qiufeng Zeng, Shiping Bai, Keying Zhang, Gang Jia

**Affiliations:** a Key Laboratory for Animal Disease-Resistance Nutrition of China Ministry of Education, Institute of Animal Nutrition, Sichuan Agricultural University, Chengdu, 611130, Sichuan, China; b Sichuan Grassland Technology Research and Promotion Center, Chengdu, 611130, Sichuan, China

**Keywords:** Microbial-phytase co-fermented sorghum, Meat duck, Nutrient utilization, Growth performance

## Abstract

This study evaluated the nutritional value and feeding effects of microbial-phytase co-fermented sorghum (MPCFS) in Cherry Valley meat ducks. MPCFS was prepared by fermentation with *Bacillus subtilis, Candida utilis*, and phytase. In experiment 1, 128 male ducks (22 days old) received a basal diet, diets replacing 30% of the basal diet with sorghum or MPCFS, or a nitrogen free diet to assess nutrients digestibility. In experiment 2, 240 male ducks (15 days old) were fed for 21 days with either a corn soybean meal diet or diets in which 70% of corn was substituted with sorghum or MPCFS. The results indicated that MPCFS significantly increased crude protein (CP), acid soluble protein (ASP), and ash in sorghum, while reducing phytic acid (PA) and fiber contents (*P* < 0.05). Moreover, MPCFS significantly improved the digestibility of CP, ether extract (EE), zinc (Zn), phosphorus (P), and gross energy (GE) (*P* < 0.05), alongside increasing the apparent metabolizable energy (AME) and the standardized ileal digestibility (SID) of Leu, Lys, Met, Val, His, and Trp (*P* < 0.05). In Experiment 2, compared with the sorghum group, the MPCFS diet significantly increased 35 day body weight (BW) and average daily gain (ADG), and decreased the feed-to-gain ratio (F/G) (*P* < 0.05). Furthermore, substituting corn with MPCFS significantly enhanced duodenal amylase, lipase and trypsin activities (*P* < 0.05). In the cecum, compared with the sorghum, MPCFS significantly increased cecal acetate, propionate, and butyrate concentrations (*P* < 0.05). Microbial analysis revealed that MPCFS improved cecal microbial α diversity in meat ducks, increased the relative abundance of Firmicutes and *Phascolarctobacterium*, and decreased that of Fusobacteriota, Bacteroidota, *Desulfovibrio*, and *Streptococcus* (*P* < 0.05). Collectively, MPCFS improved sorghum utilization in meat ducks by enhancing nutrient digestibility, digestive function, cecal short-chain fatty acid production, and gut microbial composition.

## Graphical abstract

**Figure:**
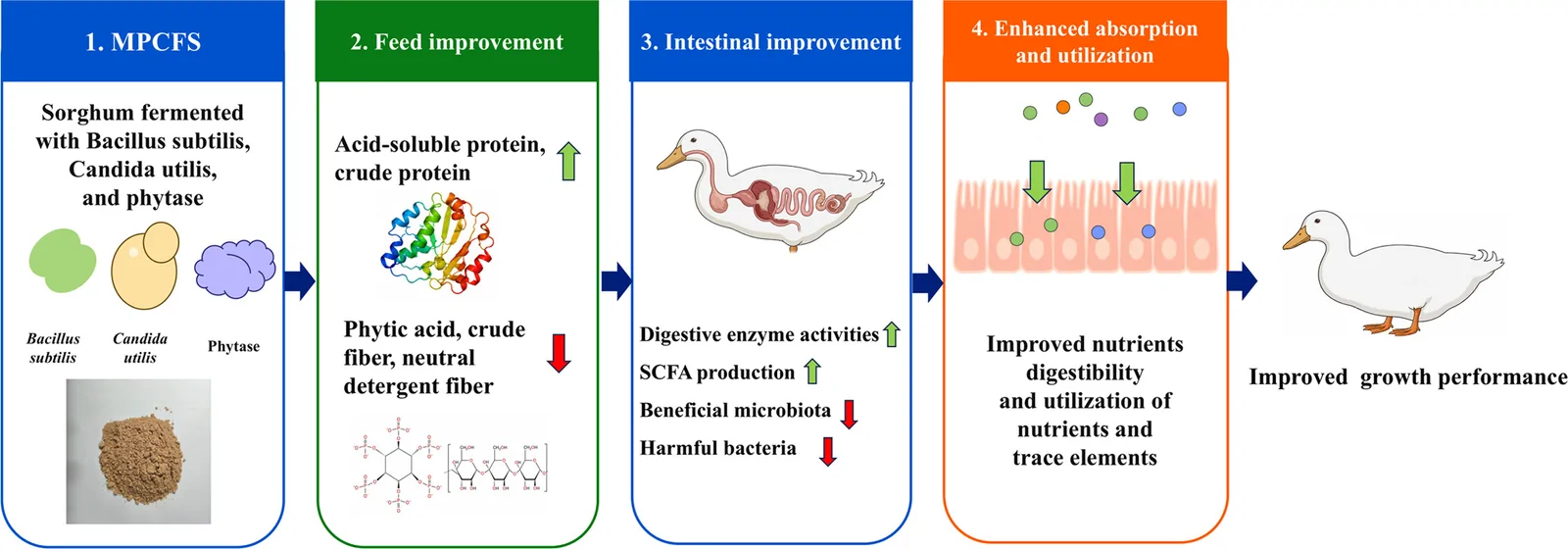

## Introduction

Sorghum ranks among the most important cereal crops globally, with extensive cultivation in Asia, the Americas, and Africa (Silva et al., 2022). Global sorghum production has been reported to reach approximately 60 million metric tons. Sorghum contains approximately 60 – 65% starch, conferring a high energy value and making it an important material for liquor fermentation as well as an energy feed ingredient in livestock and poultry diets (Selle et al., 2018; Zheng et al., 2023).

Despite its potential as a feed ingredient, sorghum utilization is hindered by certain nutritional limitations. The nutritional utilization of sorghum is restricted by antinutritional components, primarily phytic acid (PA). By forming chelates with essential divalent cations (such as zinc, calcium, magnesium, and iron) and binding to proteins, PA thereby impairs their overall bioavailability (Zhang et al., 2025). In addition, PA bound phosphorus is poorly utilized by monogastric animals, resulting in increased fecal phosphorus excretion and potential environmental pollution (Schons et al., 2011). Kafirin is another key antinutritional component affecting sorghum utilization. As the principal storage protein in sorghum grain, kafirin possesses a highly compact structure and can encapsulate or associate with starch granules, thereby reducing the accessibility of digestive enzymes and limiting nutrient hydrolysis (Wong et al., 2010; Zhang et al., 2025). Consequently, formulating diets with excessive proportions of sorghum risks suppressing animal growth and increasing the feed to gain ratio (F/G), thereby limiting overall economic and production efficiency (Batonon et al., 2015; Liu et al., 2025). Therefore, reducing antinutritional factors and improving nutrient availability are essential for enhancing the feeding value of sorghum.

Microbial-enzymatic co-fermentation (MECF) has emerged as an effective bioprocessing strategy for improving the nutritional quality of feed ingredients. This approach uses microbial metabolism and added enzymes to speed up substrate breakdown, nutrient release, and the removal of antinutritional factors (Li et al., 2021; Xu et al., 2025). During fermentation, microorganisms can produce organic acids, hydrolytic enzymes, small peptides, and other bioactive metabolites, which may improve the fermentation environment, modify substrate structure, and enhance feed palatability (Shen et al., 2024). Meanwhile, enzyme preparations can be used to degrade specific antinutritional factors or complex nutrient fractions, thereby improving nutrient availability and utilization efficiency (Shen et al., 2024; Xu et al., 2025). Previous studies have demonstrated that MECF can increase the crude protein (CP), reduce the levels of antinutritional factors such as crude fiber (CF), and limit the growth of pathogenic bacteria, including *Escherichia coli* (Du et al., 2023; Pang et al., 2024; Zhang et al., 2024). Furthermore, diets prepared by MECF can improve growth performance and nutrient utilization in pigs and poultry, as indicated by increased average daily gain (ADG), reduced F/G, and improved apparent digestibility of CP, CF, and phosphorus (P) (Zhang et al., 2026; Jia et al., 2025; Ma et al., 2025). Schons et al. reported that treating sorghum with *Paecilomyces variotii*, tannase, and phytase reduced its total phenols, hydrolyzable tannins, and condensed tannins (Schons et al., 2012). These results suggest that MESF has potential as an effective strategy to reduce antinutritional factors in sorghum and improve phosphorus utilization.

However, studies on the application of MECF to sorghum and its subsequent evaluation in meat ducks remain relatively limited. Therefore, the present study hypothesized that microbial-phytase co-fermented sorghum (MPCFS) could reduce the antinutritional factors of sorghum and improve its nutritional value for meat ducks. Accordingly, we systematically evaluated the nutritional composition of MPCFS and further conducted a feeding trial to determine its effects on growth performance, nutrient digestibility, digestive enzyme activities, cecal microbiota, and short-chain fatty acid (SCFA) profiles in meat ducks.

## Materials and methods

This study was conducted at the Research Base of Sichuan Agricultural University (Yaan, China). The Animal Welfare Committee of Sichuan Agricultural University approved animal feeding and management protocols for this study (No: 20250618).

### Preparation of microbial-phytase co-fermented sorghum (MPCFS)

Sorghum was obtained from Chifeng, a drought and semi-arid city in northern China. MPCFS was prepared by solid state fermentation using mixed cultures of *Bacillus subtilis* and *Candida utilis* at 1 × 10⁸ CFU/mL each (1:1). The water to sorghum ratio was 0.8, total inoculum was 16.0% (v/w), phytase was added at 4 U/g, and fermentation was conducted in one way valve bag at 35.0 °C for 5.0 days.

### Experiment design

Exp. 1: A total of 128 male Cherry Valley ducks (aged 22 days) were used to determine the digestibility of MPCFS and sorghum. The experimental design involved randomly assigning the ducks into 4 dietary groups, with each treatment containing 8 replicates of 4 ducks. The experimental groups consisted of: (1) basal diet (CON); (2) sorghum diet, in which 30% of the basal diet was replaced with sorghum; (3) MPCFS diet, in which 30% of the basal diet was replaced with MPCFS; and (4) a nitrogen-free diet (N-free) formulated to calculate basal endogenous amino acid losses. As an exogenous indicator, 0.5% titanium dioxide (TiO₂) was incorporated into all diets. Detailed dietary components are shown in Supplementary Tables 1 and 2.

To achieve complete gastrointestinal clearance prior to sampling, the ducks underwent a 36-h fasting period with free access to drinking water. A 72-h dietary adaptation preceded a 72-h total excreta collection period, with all acquired samples stored at −20 °C. Upon completion of the excreta collection, one healthy duck from each replicate was humanely sacrificed via carbon dioxide inhalation. Digesta from the ileum was immediately harvested into sterile 10 mL tubes and frozen at −80 °C. The apparent ileal digestibility (AID) values for amino acids were quantified utilizing the TiO₂ marker. Subsequently, the standardized ileal digestibility (SID) was derived by adjusting the AID values using the basal endogenous amino acid secretions determined from the N-free group. The calculation equations were as follows:

(1) $$Apparent\;nutrient\;digestibility\;(\%)=\left[1-\left(\frac{TiO_{2,diet}}{TiO_{2,excreta}}\right)\times \left(\frac{N_{excreta}}{N_{diet}}\right)\right]\times \;100\%$$

(2) $$AME\;(\text{MJ}/\text{kg})=GE_{diet}-\frac{GE_{excerta}\times TiO_{2,diet}}{TiO_{2,excreta}}$$

(3) $$AID_{AA}\;(\%)=\left[1-\left(\frac{TiO_{2,diet}}{TiO_{2,ileal\;digesta}}\right)\times \left(\frac{AA_{ileal\;digesta}}{AA_{diet}}\right)\right]\times \;100\%$$

(4) $$SID_{AA}\;(\%)=AID_{AA}+\frac{AA_{endogenous}}{AA_{diet}}\times \;100\%$$

Abbreviations: TiO₂, titanium dioxide; N_diet_: nutrient concentration in diet; N_excreta_: nutrient concentration in excreta; GE, gross energy; AME, apparent metabolizable energy; AA, amino acids; AID_AA_: apparent ileal digestibility of amino acids; AA_diet_: amino acid concentration in diet; AA_ileal digesta_: amino acid concentration in ileal digesta; SID_AA_: standardized ileal digestibility of amino acids; AA_endogenous_: endogenous amino acid losses.

Exp. 2: 240 ducks with similar body weights were selected and randomly allocated to three dietary treatments: (1) a basal diet group (control); (2) a sorghum group, in which sorghum replaced 70% of the corn in the basal diet; and (3) an MPCFS group, in which MPCFS replaced 70% of the corn. Each treatment consisted of 8 replicates (10 ducks per replicate). The diet formulations for day 15 to 35 are shown in Table 1. Nutrient levels were formulated according to the Chinese Standard for Nutrient Requirements of Meat Ducks (GB/T 45103-2024).

**Table 1:** Composition and nutrient level of the experiment 2 diet (15-35d, air-dry basis, %).

Items	Control	Sorghum	MPCFS
Corn	61.00	18.30	18.30
Soybean meal	26.98	25.54	25.84
Soybean oil	2.00	2.50	2.20
Wheat Bran	6.00	7.00	7.00
Sorghum	0.00	42.70	0.00
MPCFS	0.00	0.00	42.70
L-Lys-HCl, 98.5%	0.07	0.09	0.09
L-Thr, 98.5%	0.05	0.08	0.08
Trp	0.02	0.02	0.02
L-Met, 99%	0.18	0.19	0.19
CaCO₃	0.90	1.05	1.05
CaHPO₄	1.77	1.50	1.50
NaCl	0.35	0.35	0.35
Choline chloride, 50%	0.15	0.15	0.15
Vitamin premix^1^	0.03	0.03	0.03
Mineral premix^2^	0.50	0.50	0.50
Total	100.00	100.00	100.00
Nutrient levels^3^, %			
ME, MJ/kg	12.14	12.12	12.10
CP	17.50	17.49	17.59
Ca	0.85	0.85	0.85
Lys	0.85	0.90	0.90
Met	0.40	0.41	0.41
Trp	0.18	0.19	0.19
Thr	0.60	0.62	0.62

1 Vitamin premix provides the following per kg of diets: Vitamin A 3,000 IU, Vitamin D_3_ 2,000 IU, Vitamin E 10 IU, Vitamin B_1_ 1.5 mg, Vitamin B_2_ 10 mg, Vitamin B_5_ 11 mg, Vitamin K_3_ 2 mg, Vitamin B_6_ 3 mg, Vitamin B_9_ 1 mg, Vitamin B_12_ 0.02 mg, and Vitamin B_3_ 50 mg.

2 Mineral premix provides the following per kg of the diet: Fe 60.0 mg, Se 0.3 mg, Mn 80.0 mg, I 0.3 mg, Cu 8.0 mg, Zn 60.0 mg.

3 Nutrient levels are calculated values.

### Sample collection and data recording

On day 35, following a 12-hour period of feed withdrawal, the body weight of all birds was recorded. From each pen, a single representative duck exhibiting a body weight closest to the average pen weight was chosen for sampling (n = 8). Jugular blood samples were drawn, and the serum was subsequently extracted via centrifugation (3,000 × *g*, 4 °C, 15 min). After the ducks were euthanized, luminal contents from the duodenum and cecum were directly collected into 5-mL sterile tubes, snap-frozen in liquid nitrogen, and stored at −80 °C until analysis.

### Chemical composition

Dry matter (DM), crude protein (CP), ether extract (EE), ash, phosphorus (P), zinc (Zn), and calcium (Ca) were analyzed following the standard methods of AOAC (2023). Crude fiber (CF), neutral detergent fiber (NDF), and acid detergent fiber (ADF) were analyzed utilizing an ANKOM 2000 Fiber Analyzer (ANKOM Technology, USA). Measurement of gross energy (GE) was conducted using an oxygen bomb calorimeter (6400, Parr Instrument Company, USA). Additionally, amino acid profiles were analyzed using an automatic amino acid analyzer (L-8900, Hitachi High-Technologies Corporation, Japan). Phytic acid (PA) and acid-soluble protein (ASP) levels in feed ingredients were determined in accordance with GB 5009.153-2016 and GB/T 22492-2008, respectively. Titanium dioxide (TiO₂) concentrations in feed ingredients and excreta were measured using the method described by Short et al. (1996).

### Serum biochemistry

Serum biochemical profiles were quantified utilizing an automatic biochemical analyzer (7080ISE, Hitachi High-Technologies Corporation, Japan). The evaluated parameters consisted of lipid profiles, including total cholesterol (TC), triglycerides (TG), high-density lipoprotein cholesterol (HDL-C), and low-density lipoprotein cholesterol (LDL-C); protein and nitrogenous metabolites, such as total protein (TP), albumin (ALB), blood urea nitrogen (BUN), and uric acid (UA); hepatic enzymes, namely alanine aminotransferase (ALT) and aspartate aminotransferase (AST); as well as glucose (GLU).

### Digestive enzyme activity

To evaluate digestive enzyme activities, 10% homogenates of duodenal digesta were prepared in physiological saline on ice and subsequently centrifuged at 3,000 r/min for 10 min. The resulting supernatants were harvested and assayed for the activities of amylase, lipase, and trypsin using commercial kits (A080-2-2, A054-2-1, and C0016-1-2, respectively) purchased from Nanjing Jiancheng Bioengineering Institute (China).

### Short chain fatty acids (SCFAs)

Based on the extraction method outlined by Chang et al. (2024) with slight adjustments, SCFAs were analyzed utilizing an Agilent 7890A gas chromatograph (Agilent Technologies, USA). For sample preparation, cecal digesta (approximately 0.5 g) was suspended in 1.5 mL of distilled water and centrifuged (3,000 × *g*, 4 °C, 10 min). The supernatant (1 mL) was subsequently acidified using 23.3 μL of crotonic acid and 0.2 mL of 25% metaphosphoric acid. Following a 30-minute incubation on ice and high-speed centrifugation (12,000 × g, 10 min, 4 °C), 0.3 mL of the cleared extract was combined with 0.9 mL of methanol for GC assessment.

### Cecum microbiota

The extraction of genomic DNA from cecal digesta was carried out with a CretMag™ Power Soil DNA Kit (Cretaceous, China). Following quantification (NanoDrop 2000 spectrophotometer) and quality verification (1% agarose gel electrophoresis), the purified DNA functioned as the template for PCR. The V3–V4 hypervariable region of the bacterial 16S rRNA gene was amplified using ESTaq MasterMix (Dye) and specific primers as previously described (Zhang et al., 2023). After purifying the amplified products via the AxyPrep DNA Gel Extraction Kit (Axygen Biosciences, USA), the amplicons were subjected to paired-end sequencing on an Illumina MiSeq PE300 platform (Illumina Inc., San Diego, USA) and subsequent bioinformatics evaluations by Metware Biotechnology Inc. (Wuhan, China).

### Statistical analysis

Data were analyzed with SAS 9.4. The assumptions of normal distribution and equal variance were checked using the Shapiro–Wilk and Levene’s tests, respectively. In Exp. 1, the replicate cage was considered the experimental unit for excreta based measurements, whereas the sampled duck from each replicate was used as the experimental unit for ileal digesta related measurements. Differences between sorghum and MPCFS were analyzed using Student’s t-test. In Exp. 2, the replicate cage served as the experimental unit for growth performance. For serum biochemical indices, digestive enzyme activities, cecal short-chain fatty acid concentrations, and microbiota-related measurements, the individual duck selected from each replicate cage was considered the experimental unit. Data were analyzed by one-way ANOVA using the GLM procedure of SAS 9.4, and means were separated using Tukey’s multiple comparison test when a significant treatment effect was observed. Values are reported as means ± SEM. Differences were considered significant at *P* < 0.05, and tendencies were defined as 0.05 ≤ *P* < 0.10. Pearson’s correlation coefficients were calculated to examine relationships among selected variables, and correlations with |r| ≥ 0.5 were considered meaningful.

## Results

### Chemical composition

Table 2 show the differences in the nutritional composition of sorghum and MPCFS. Compared with sorghum, MPCFS significantly improved the levels of CP, ASP, and ash (*P* < 0.05), whereas the levels of DM, CF, NDF, PA, and GE were significantly decreased (*P* < 0.05). Furthermore, no significant differences in amino acid composition were observed between sorghum and MPCFS (*P* > 0.05).

**Table 2:** The nutritional composition of Sorghum and MPCFS (Dry Matter basis).

Items	Sorghum	MPCFS	SEM	*P*-value
DM, %	89.1	87.9	0.215	0.016
CP, %	9.64	10.4	0.024	<0.001
ASP, %	0.393	3.64	0.056	<0.001
EE, %	3.45	3.41	0.035	0.404
CF, %	1.82	1.48	0.016	<0.001
NDF, %	8.09	5.95	0.112	0.003
ADF, %	4.29	4.18	0.059	0.274
ash, %	1.85	1.95	0.020	0.027
Ca, %	0.323	0.303	0.011	0.257
Zn, mg/kg	19.1	19.1	0.143	0.940
TP, %	0.251	0.257	0.003	0.954
PA, %	0.914	0.357	0.003	<0.001
GE, kcal/kg	4025	3960	6.87	0.003
Asp	0.712	0.705	0.014	0.771
Thr	0.373	0.368	0.010	0.753
Ser	0.479	0.475	0.010	0.830
Glu	2.181	2.151	0.042	0.666
Gly	0.332	0.322	0.011	0.587
Ala	1.03	1.00	0.048	0.705
Cys	0.094	0.084	0.021	0.765
Val	0.450	0.400	0.041	0.481
Met	0.107	0.100	0.014	0.759
Ile	0.426	0.415	0.008	0.498
Leu	1.59	1.56	0.030	0.608
Tyr	0.626	0.602	0.009	0.212
Phe	0.546	0.531	0.008	0.310
Lys	0.219	0.218	0.009	0.973
His	0.368	0.331	0.027	0.436
Arg	0.429	0.395	0.033	0.538
Pro	1.08	1.06	0.022	0.555
Trp	0.084	0.085	0.001	0.824

Note: Data are presented as means with the pooled SEM. (n = 3). DM, dry matter; CP, crude protein; ASP, acid-soluble protein; EE, ether extract; CF, crude fiber; NDF, neutral detergent fiber; ADF, acid detergent fiber; Ca, calcium; Zn, zinc; TP, total phosphorus; PA, phytic acid; GE, gross energy.

### Nutrient digestibility and ileal amino acid digestibility

Table 3, Table 4 show the nutrients and amino acid digestibility of sorghum and MPCFS in meat ducks. MPCFS significantly increased the apparent digestibility of CP, EE, ash, Zn, P, and GE in sorghum (*P* < 0.05). Moreover, the AME of sorghum in meat ducks was significantly improved after fermentation (*P* < 0.05). MPCFS significantly increased both the AID and SID of leucine, lysine, methionine, valine, histidine, and tryptophan, as well as those of the tyrosine, in meat ducks (*P* < 0.05).

**Table 3:** Nutrient utilization of sorghum and MPCFS in meat ducks in Experiment 1 (dry matter basis, %).

Items	Nutrient utilization			
	Sorghum	MPCFS	SEM	*P*-value
DM, %	73.20	76.4	0.923	0.121
CP, %	63.5	73.7	2.15	0.029
CF, %	14.1	16.1	0.503	0.096
EE, %	77.1	82.9	0.595	0.002
NDF, %	16.4	18.7	1.92	0.464
ADF, %	12.4	13.3	1.27	0.627
ash, %	31.8	39.5	0.673	0.001
Zn, %	23.5	34.9	0.747	0.002
Ca, %	43.0	46.39	1.01	0.089
P, %	31.5	40.8	0.946	0.016
GE, %	77.8	85.3	0.681	0.001
AME, MJ/kg	14.0	14.6	0.403	0.002

Note:Data are presented as means with the pooled SEM. (n = 8).

**Table 4:** Amino acid digestibility of sorghum and MPCFS in meat ducks in Experiment 1 (dry matter basis, %).

Item	Apparent ileal digestibility		SEM	*P*-value	Standardized ileal digestibility		SEM	*P*-value
	Sorghum	MPCFS			Sorghum	MPCFS		
EAAs								
Arg	75.8	76.9	1.15	0.532	86.7	86.8	0.409	0.860
Thr	67.2	69.1	0.193	0.153	72.9	74.4	0.549	0.107
Leu	84.4	87.0	0.318	0.005	87.2	89.4	0.285	0.006
Phe	84.3	84.1	0.285	0.755	86.6	85.0	0.540	0.097
Lys	67.2	71.1	0.357	0.002	76.2	79.0	0.378	0.007
Ile	77.0	73.7	0.411	0.507	75.3	77.3	0.464	0.015
Met	82.3	85.4	0.538	0.015	86.0	87.1	0.269	0.045
Val	81.7	83.0	0.862	< 0.001	85.1	86.9	0.336	0.020
His	72.6	76.4	0.233	< 0.001	75.1	80.3	0.271	< 0.001
Trp	73.5	77.9	0.480	0.003	81.9	85.0	0.351	0.004
Ser	72.4	72.1	0.565	0.799	80.5	81.2	0.323	0.197
Gly	71.7	71.2	0.615	0.542	76.8	77.2	0.188	0.213
NEAAs								
Tyr	71.6	75.0	0.554	0.014	75.7	77.7	0.385	0.022
Glu	81.9	82.3	0.724	0.744	83.2	83.1	0.292	0.834
Ala	80.9	79.1	0.318	0.145	83.6	84.9	0.355	0.057
Asp	72.6	73.5	0.998	0.551	76.7	77.0	0.540	0.693
Pro	73.1	73.2	0.428	0.833	81.9	82.0	0.304	0.783
Cys	68.7	68.6	0.536	0.965	74.1	74.0	0.195	0.955

Note: Amino acid digestibility was determined using ileal digesta samples collected from one duck per replicate (n = 8). Data are presented as means with the pooled SEM. EAA, essential amino acids; NEAA, non-essential amino acids.

### Growth performance

Growth performance of meat ducks is shown in Table 5. Compared with the control and MPCFS groups, the sorghum group had significantly lower BW at 35 d and ADG from 15 to 35 d in meat ducks (*P* < 0.05). Furthermore, MPCFS significantly reduced the F/G compared with sorghum (*P* < 0.05), with no significant difference from the control group (*P* > 0.05).

**Table 5:** Effects of MPCFS on growth performance of meat ducks in Experiment 2.

Items		15∼35 days		SEM	*P*-value
	Control	Sorghum	MPCFS		
BW at 15d, g	581	581	579	1.12	0.801
BW at 35d, g	2734^a^	2610^b^	2761^a^	23.3	0.012
ADG, g	103^a^	96.7^b^	104^a^	1.09	0.010
ADFI, g	180	172	175	1.66	0.167
F/G	1.76^ab^	1.78^a^	1.68^b^	0.026	0.014

Note: Each treatment consisted of 8 replicates with 10 ducks per replicate. Data are presented as means with the pooled SEM. ^a,b^ Means within the same row that have no common superscript are significantly different at *P* < 0.05.

### Serum biochemistry

The serum biochemical indices of meat ducks are shown in Table 6. The MPCFS group exhibited significantly higher serum ALB and TP concentrations than the sorghum group (*P* < 0.05). However, no significant differences in serum ALB or TP concentrations were detected between the MPCFS and control groups (*P* > 0.05). Compared with the control group, both the sorghum and MPCFS groups had significantly lower serum TG concentrations (*P* < 0.05).

**Table 6:** Effects of dietary MPCFS on serum biochemical parameters and duodenal digestive enzyme activities of meat ducks in Experiment 2.

Items	Control	Sorghum	MPCFS	SEM	*P*-value
Serum biochemical parameters					
ALB, g/L	10.6^a^	8.01^b^	10.3^a^	0.327	0.003
ALT, U/L	50.3	52.7	48.4	2.839	0.587
AST, U/L	20.9	20.7	21.4	0.887	0.862
GLU, mmol/L	8.52	8.42	8.33	0.425	0.952
HDL-C, mmol/L	1.98	1.91	2.08	0.063	0.257
LDL-C, mmol/L	1.27	1.24	1.21	0.084	0.852
TC, mmol/L	3.69	3.66	3.69	0.061	0.934
TG, mmol/L	0.952^a^	0.720^b^	0.704^b^	0.031	0.002
TP, g/L	28.2^a^	22.6^b^	28.4^a^	1.00	0.010
BUN, mmol/L	0.396	0.398	0.399	0.007	0.968
Duodenal digestive enzyme activities					
Amylase activity, U/mg prot	0.065^b^	0.062^b^	0.073^a^	0.001	<0.001
Lipase activity, U/mg prot	127^b^	117^b^	143^a^	5.36	0.005
Trypsin activity, U/mg prot	2404^b^	2043^c^	2578^a^	34.7	0.017

Note: Each treatment consisted of 8 replicates with 1 bird sampled per replicate. Data are presented as means with the pooled SEM. ALB, albumin; ALT, alanine aminotransferase; AST, aspartate aminotransferase; GLU, glucose; HDL-C, high-density lipoprotein cholesterol; LDL-C, low-density lipoprotein cholesterol; TC, total cholesterol; TG, triglycerides; TP, total protein; BUN, blood urea nitrogen. ^a,b^ Means within the same row that have no common superscript are significantly different at *P* < 0.05.

### Duodenal digestive enzymes

The MPCFS group showed significantly higher duodenal amylase, lipase, and trypsin activities than the control and sorghum groups (*P* < 0.05, Table 6).

### Short chain fatty acids (SCFAs)

Table 7 presents the data on SCFAs. Compared with the sorghum group, ducks fed either the control or MPCFS diets exhibited markedly increased levels of cecal acetate, propionate, and butyrate (*P* < 0.05). Furthermore, the MPCFS resulted in a significantly greater concentration of cecal butyrate compared to the control group (*P* < 0.05).

**Table 7:** Effects of MPCFS on cecal short chain fatty acids concentrations and α-diversity of cecal microbiota in meat ducks in Experiment 2.

Items	Control	Sorghum	MPCFS	SEM	*P*-value
Short chain fatty acids					
AA, mg/g	3.50^a^	3.27^b^	3.50^a^	0.022	<0.001
PA, mg/g	1.18^a^	1.07^b^	1.21^a^	0.019	<0.001
IBA, mg/g	0.014	0.013	0.016	0.003	0.729
BA, mg/g	0.603^b^	0.513^c^	0.697^a^	0.018	0.001
IVA, mg/g	0.040	0.033	0.040	0.005	0.593
VA, mg/g	0.137	0.127	0.123	0.009	0.954
α-diversity of cecal microbiota					
ACE	445	434	454	6.99	0.547
Chao1	455	435	458	7.40	0.440
Shannon	4.06^b^	3.95^b^	4.34^a^	0.067	0.005
Observed-ASV	361^b^	403^ab^	428^a^	11.3	0.018

Note: Data are presented as means with the pooled SEM. cecal short-chain fatty acid concentrations were determined using n = 8, whereas cecal microbiota α-diversity was analyzed using n = 5. AA, acetic acid; PA, propionic acid; IBA, isobutyric acid; BA, butyric acid; IVA, isovaleric acid; VA, valeric acid. ^a,b^ Means within the same row that have no common superscript are significantly different at *P* < 0.05.

### Cecal microbiota

**Fig. 1:**
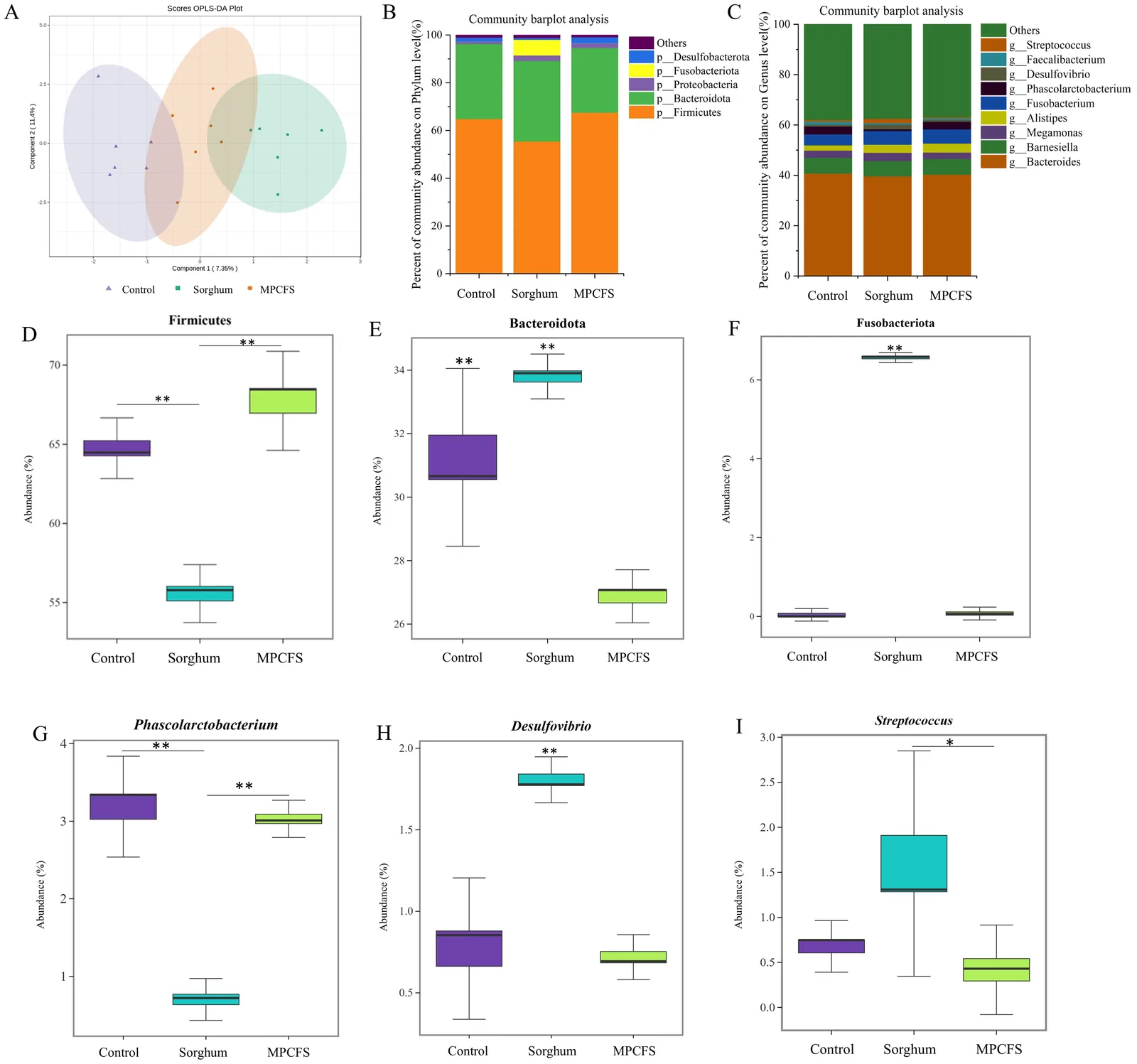
Effects of MPCFS on the cecal microbiota of meat ducks. Data are expressed as the mean ± SEM. * indicates *P* < 0.05, ** indicates *P* < 0.01.

Regarding cecal microbial α diversity (Table 7), the MPCFS group exhibited a significantly higher Shannon index than both the control and sorghum groups (*P* < 0.05), alongside an increased Observed ASVs index compared to the control (*P* < 0.05). The OPLS-DA results showed a certain separation trend in the cecal microbial community structure among the three groups (Fig. 1A). The control group was mainly distributed in the negative region of Component 1, whereas the sorghum group was clearly separated into the positive region. The MPCFS group clustered between them, showing partial overlap with both groups and displaying an intermediate structural pattern.

At the phylum level (Fig. 1B), Firmicutes and Bacteroidota predominated in the cecal microbial communities of meat ducks. The relative abundance of Firmicutes was markedly higher in the control and MPCFS groups than in the sorghum group (*P* < 0.05; Fig. 1D), while Fusobacteriota showed the opposite trend, with significantly lower abundance in these two groups (*P* < 0.05; Fig. 1F). Notably, MPCFS supplementation led to a significant reduction in Bacteroidota abundance compared with both the control and sorghum groups (*P* < 0.05; Fig. 1E). At the genus level (Fig. 1C), Bacteroides was identified as the predominant genus in the cecal microbiota. The sorghum group exhibited a higher abundance of Desulfovibrio and a lower abundance of Phascolarctobacterium than the control and MPCFS groups (*P* < 0.05; Fig. 1G,H). Furthermore, Streptococcus abundance was significantly decreased in the MPCFS group compared with the sorghum group (*P* < 0.05; Fig. 1I).

### Correlation analysis

**Fig. 2:**
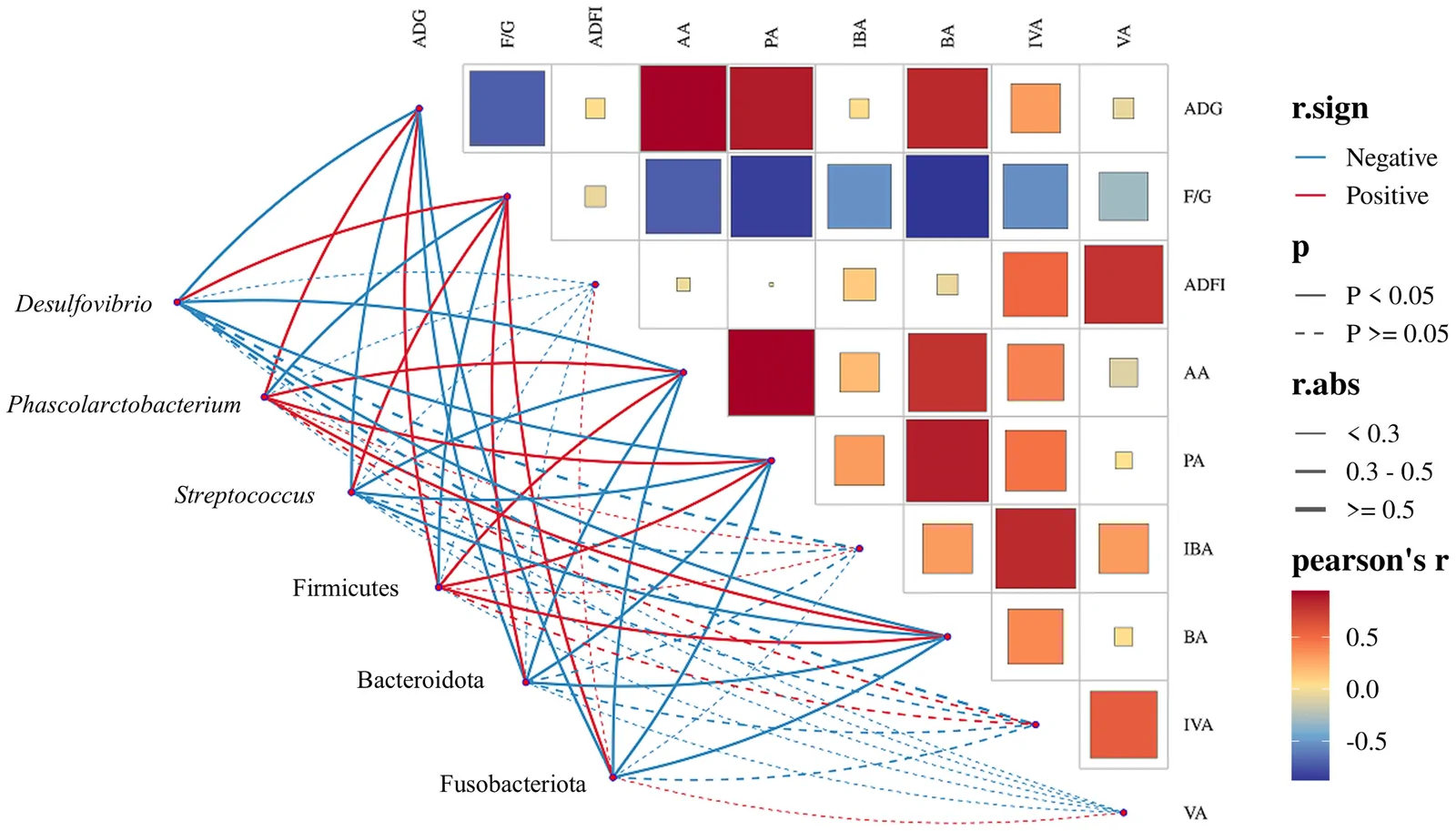
Correlation analysis among growth performance, cecal SCFAs, and cecal microbiota in meat ducks.

Pearson correlation analysis demonstrated that specific microbiota were significantly associated with growth performance and SCFAs (Fig. 2). Firmicutes and *Phascolarctobacterium* exhibited a strong positive correlation with ADG and SCFAs (AA, PA, and BA), while showing a negative correlation with F/G (*P* < 0.05, |r| > 0.5). In contrast, Bacteroidota, Fusobacteriota, *Desulfovibrio* and *Streptococcus* were predominantly negatively correlated with ADG and SCFAs, while showing a positive correlation with F/G (*P* < 0.05, |r| > 0.5).

## Discussion

Extensive evidence demonstrates that MECF not only upgrades feedstuff quality through the reduction of antinutritive substances but also significantly augments the bioavailability of essential nutrients (Lin et al., 2020; Xu et al., 2026). In the present study, MPCFS increased CP and ASP contents, whereas DM, CF, NDF, GE, and PA contents were significantly decreased, indicating that MPCFS improved the nutritional value of sorghum. The microbial depletion of carbohydrates leads to a proportional enrichment of existing nutrients and, combined with the continuous buildup of microbial cell proteins, drives the observed enhancement in CP content (Chen et al., 2021; Liu et al., 2026). Meanwhile, macromolecular proteins are broken down by proteases from fermenting microorganisms into small peptides and ASP, which exhibit enhanced digestibility and absorption (Shi et al., 2017; Chen et al., 2021). In addition, hydrolytic enzymes such as cellulases can disrupt plant cell wall structures and the intermolecular linkages of fibrous components, thereby promoting fiber degradation (Su et al., 2021). Moreover, the consumption of fermentable carbohydrates, such as starch, by microorganisms during growth and proliferation may also contribute to the reductions in DM and GE contents.

In the present study, phytate content in MPCFS decreased by 61.54% compared with untreated sorghum. Phytate degradation may not only weaken its binding capacity with minerals and proteins, but also release phytate bound phosphorus, thereby improving the bioavailability of phosphorus and other minerals (Schlemmer et al., 2009). Consistent with this, MPCFS significantly increased the apparent digestibility of CP, EE, ash, Zn, P, and GE, as well as the AME of sorghum in meat ducks. These results indicate that MPCFS improved nutrient availability in addition to modifying the chemical composition of sorghum. Notably, the increased digestibility of P and Zn was likely associated with phytate degradation and the release of bound minerals (Singh, 2008; Maria et al., 2024). Moreover, both phytate and kafirin in sorghum may limit the release and absorption of amino acids (Zouaoui et al., 2018). The current results demonstrate that MPCFS substantially elevated both the AID and SID of key amino acids (e.g., lysine, methionine, and tryptophan). These beneficial effects are likely attributable to the concurrent degradation of phytate and structural shifts within the sorghum protein matrix. Importantly, this aligns with our previous findings that fermentation alters the structural matrix of sorghum, creating larger pores that enhance contact with digestive enzymes (Liu et al., 2026). Therefore, MPCFS may improve the nutritional composition and nutrient utilization of sorghum by reducing antinutritional factors such as phytate and fiber, and by enhancing its digestive accessibility.

Given the improvements in nutrient composition and digestibility of MPCFS, the impact of substituting corn with MPCFS on the growth performance of meat ducks was further evaluated. Consistent with previous studies, the present research demonstrated that replacing corn with sorghum adversely affected the productive performance of meat ducks (Batonon et al., 2015; Liu et al., 2025), significantly decreasing the BW at 35 days of age and the ADG from days 15 to 35, while increasing the F/G. The restriction of nutrient liberation by kafirin, fiber, and PA is the probable cause of the observed growth depression in sorghum fed ducks. However, the MPCFS process appears to effectively dismantle these antinutritional barriers. This is evidenced by the MPCFS group significantly decreased F/G compared to the sorghum group. Furthermore, ducks fed the MPCFS diet exhibited significantly elevated serum levels of TP and ALB relative to the sorghum group. Since TP and ALB are closely associated with protein and amino acid metabolism (Zhao et al., 2021; Son et al., 2024), these results indicate that fermentation enhanced the bioavailability and utilization of proteins and amino acids. Furthermore, MPCFS significantly increased the activities of amylase, lipase, and trypsin in the duodenum. This stimulatory effect is presumably attributable to the microbial breakdown of antinutritional components, such as phytic acid and tannins, which effectively alleviated their inhibitory effects on digestive enzymes (Liu et al., 2009; Jiang et al., 2020). Furthermore, the generation of fermentation by-products, including small peptides and organic acids, may have provided additional stimuli for enzyme secretion (Karimzadeh et al., 2016; Ma et al., 2021). Consistent with these findings, the MPCFS group exhibited significantly increased cecal concentrations of acetate, propionate, and butyrate in this study. SCFAs, particularly butyrate, may enhance digestive enzyme activity by improving intestinal morphology, modulating luminal pH and stimulating pancreatic exocrine secretion (Wu et al., 2018). Thus, the improved growth performance observed in meat ducks fed MPCFS may be attributed to the reduced antinutritional factors, enhanced digestive enzyme activities, increased SCFA production, and improved protein and amino acid utilization. Overall, MPCFS improved nutrient utilization and digestive function by reducing antinutritional factors, enhancing digestive enzyme activities, and increasing SCFA production, thereby supporting the growth performance of meat ducks.

The intestinal microbiota is closely associated with nutrient utilization and gut health. Therefore, we further analyzed the cecal microbial community to clarify the potential microbial mechanisms underlying the effects of MPCFS. In the present study, MPCFS increased the Shannon index and observed ASV index, indicating that fermented sorghum enhanced the diversity and richness of the cecal microbiota. This increase in microbial diversity may reflect a more resilient intestinal ecosystem with greater metabolic potential for substrate utilization (Lozupone et al., 2012; Fassarella et al., 2021). The microbial community was primarily dominated by the phylum Firmicutes. MPCFS group increased the abundance of Firmicutes and decreased that of Fusobacteriota and Bacteroidota. Firmicutes includes a variety of bacterial taxa involved in carbohydrate fermentation and SCFA production, and its increased abundance may therefore contribute to the elevated levels of acetate, propionate, and butyrate (Qin et al., 2020). In contrast, an increase in Fusobacteriota is generally associated with a greater risk of intestinal microbial dysbiosis (Gu et al., 2020). As Bacteroidetes are specialized in degrading and utilizing complex dietary polysaccharides (Thomas et al., 2011), their reduced abundance in the MPCFS group may be associated with the partial degradation of fiber and complex carbohydrates in sorghum during fermentation. Moreover, the expansion of Firmicutes may also contribute to the relative decrease in Bacteroidetes, given that microbial community data are compositional. At the genus level, compared with the sorghum group, MPCFS increased the abundance of *Phascolarctobacterium* and decreased the abundances of *Desulfovibrio* and *Streptococcus. Phascolarctobacterium* is capable of producing SCFAs, such as acetate and propionate, and its abundance is associated with host metabolic status (Wu et al., 2017). Conversely, *Desulfovibrio* is a sulfate reducing bacterium whose excessive enrichment can increase hydrogen sulfide production, leading to intestinal inflammation or barrier dysfunction (Singh et al., 2023; Huang et al., 2024). Similarly, previous studies have implicated *Streptococcus* in the pathogenesis of multiple poultry diseases, including septicemia, endocarditis, and arthritis (Gray et al., 2023). Collectively, the enrichment of potential SCFA producing bacteria alongside the reduction of potentially harmful and pathogenic taxa suggests that MPCFS improves the intestinal health and microbial stability of meat ducks.

To further elucidate the intrinsic link between cecal microbiota succession and host phenotypes, a correlation analysis was conducted. We observed that potentially beneficial, SCFA producing microbiota (Firmicutes and *Phascolarctobacterium*) were strongly and positively correlated with cecal SCFA concentrations and ADG, while negatively correlated with the F/G ratio. Conversely, Bacteroidota, Fusobacteriota, *Desulfovibrio*, and *Streptococcus* exhibited negative correlations with growth performance and SCFAs. This suggests that the improvement in feed efficiency and growth performance induced by MPCFS may be associated with alterations in the cecal microbiota and enhanced SCFA production. These microbial shifts could be a byproduct of the improved gut environment or a direct driver of host metabolism. Future studies utilizing multi-omics, fecal microbiota transplantation, or specific strain colonization are required to verify their exact causal roles.

## Conclusions

In conclusion, MPCFS enhances the nutritional value of sorghum by degrading anti-nutritional factors such as phytic acid and fiber. Furthermore, dietary inclusion of MPCFS effectively alleviates the adverse effects of raw sorghum on meat duck growth, improves digestive enzyme activities, increases cecal SCFA production, and modulates the cecal microbiota. These findings provide a theoretical basis for the application of fermented sorghum as an alternative energy feed ingredient in animal production.

## Ethics approval

This study was approved by the Animal Welfare Committee of Sichuan Agricultural University (No: 20250618).

## Author Contribution

Junlei Chang: Completed the core experimental design of the study, conducted the main animal experiments and experimental investigation, performed animal slaughter and sample collection, conducted the primary data sorting and analysis, drafted the original manuscript, and completed the figure production and data visualization. Yu Deng: Participated in the formulation of the experimental schemes, took part in the main animal experimental investigation, assisted in animal slaughter and sample collection, and sorted, organized, and processed the experimental data. Jianqi Yang: Participated in the development and refinement of the analytical framework, conducted formal analysis of the core datasets, interpreted the key research findings, and contributed to manuscript revision and scientific discussion. Yang Liu, Leizheng Zhang, Dongmei Xie, and Xuemei Ding: Participated in the experimental design and investigation, conducted animal slaughter and sample collection, performed sample processing and relevant experimental measurements, and contributed to the sorting and analysis of experimental data. Jianping Wang, Yan Liu, and Qiufeng Zeng: Participated in the formulation of experimental schemes, conducted corresponding animal experimental investigations and sample collection, assisted in the organization and verification of experimental data, and contributed to the interpretation of research findings and scientific discussion. Shiping Bai and Keying Zhang: Participated in the development and refinement of the analytical framework, conducted relevant data processing and analysis, contributed to the interpretation and verification of research findings, and assisted in manuscript revision. Gang Jia: Conceived the original research idea and overall research framework, provided academic and methodological guidance for the study, supervised the progress and quality of the research, interpreted the main research findings, and critically revised the manuscript.

## Financial support statement

This study was supported by the 10.13039/501100012166National Key Research and Development Program of China (2023YFD1301402) and 10.13039/501100013314Higher Education Discipline Innovation Project of China (D17015).

## Disclosures

The authors declare that there is no conflict of interest.

## Data Availability

Data sharing is not applicable to a public repository for this study and the relevant datasets are available upon request.

## Appendix Supplementary materials

Image, application 1

## Footnotes

Supplementary material associated with this article can be found, in the online version, at doi:10.1016/j.psj.2026.107526.
