# Supplementary material for: Effects of microbial-phytase co-fermentation of sorghum on growth performance, nutrient utilization, digestive enzyme activities and cecal microbiota in meat ducks
Source: Poult Sci. 2026 Jul 30;105(11):107526. doi: 10.1016/j.psj.2026.107526 (PMC13542589; doi:10.1016/j.psj.2026.107526)
Supplement: Supplementary file 1 [file mmc1.docx]

**Table 1. Composition and nutrient levels of experimental 1 diet (Dry Matter basis).**

| **Items** | **CON** | **Sorghum** | **MPCFS** |
| --- | --- | --- | --- |
| Ingredients, % |  |  |  |
| Corn | 61.80 | 32.60 | 32.60 |
| Soybean meal | 26.64 | 25.88 | 25.88 |
| Soybean oil | 2.00 | 2.00 | 2.00 |
| Wheat Bran | 5.00 | 5.00 | 5.00 |
| Sorghum | 0.00 | 30.00 | 0.00 |
| MESFS | 0.00 | 0.00 | 30.00 |
| L-Lys-HCl, 98.5% | 0.07 | 0.09 | 0.09 |
| L-Thr, 98.5% | 0.04 | 0.05 | 0.05 |
| Trp | 0.02 | 0.01 | 0.01 |
| L-Met, 99% | 0.17 | 0.17 | 0.17 |
| CaCO_3_ | 1.14 | 1.02 | 1.02 |
| CaHPO_4_ | 1.59 | 1.65 | 1.65 |
| NaCl | 0.35 | 0.35 | 0.35 |
| Choline chloride | 0.15 | 0.15 | 0.15 |
| Vitamin premix ^1^ | 0.03 | 0.03 | 0.03 |
| Mineral premix ^2^ | 0.50 | 0.50 | 0.50 |
| TiO_2_ | 0.50 | 0.50 | 0.50 |
| Total | 100.00 | 100.00 | 100.00 |
| Nutrient levels ^3^, % |  |  |  |
| Dry Matter | 86.49 | 87.06 | 86.56 |
| Crude protein | 17.52 | 17.53 | 17.71 |
| Calcium | 0.85 | 0.85 | 0.85 |
| Available phosphorus | 0.40 | 0.40 | 0.40 |

^1^ Vitamin premix provides the following per kg of diets: Vitamin A 3,000 IU, Vitamin D_3_ 2,000 IU, Vitamin E 10 IU, Vitamin B_1_ 1.5 mg, Vitamin B_2_ 10 mg, Vitamin B_5_ 11 mg, Vitamin K_3_ 2 mg, Vitamin B_6_ 3 mg, Vitamin B_9_ 1 mg, Vitamin B_12_ 0.02 mg, and Vitamin B_3_ 50 mg.

^2^ Mineral premix provides the following per kg of the die: Fe 60.0 mg, Se 0.3 mg, Mn 80.0 mg, I 0.3 mg, Cu 8.0 mg, Zn 60.0 mg.

^3^ Nutrient levels are calculated values.

**Table 2. Composition and nutrient levels of the nitrogen-free diets (Dry Matter basis).**

| **Items** | **Content** |
| --- | --- |
| Ingredients, % |  |
| Corn starch | 70.80 |
| Glucose | 21.24 |
| Carboxymethylcellulose | 3.00 |
| CaCO_3_ | 0.51 |
| CaHPO_4_ | 2.33 |
| NaHCO_3_ | 0.54 |
| KCl | 0.25 |
| K_2_SO_4_ | 0.20 |
| Cholinechloride | 0.10 |
| Vitamin premix ^1^ | 0.03 |
| Mineral premix ^2^ | 0.5 |
| TiO_2_ | 0.5 |
| Total | 100 |
| Nutrient levels ^3^, % |  |
| Metabolizable Energy, MJ/kg | 2850 |
| Crude protein | 0.28 |
| Calcium | 0.85 |
| Available phosphorus | 0.40 |

^1^ Vitamin premix provides the following per kg of diets: Vitamin A 3,000 IU, Vitamin D_3_ 2,000 IU, Vitamin E 10 IU, Vitamin B_1_ 1.5 mg, Vitamin B_2_ 10 mg, Vitamin B_5_ 11 mg, Vitamin K_3_ 2 mg, Vitamin B_6_ 3 mg, Vitamin B_9_ 1 mg, Vitamin B_12_ 0.02 mg, and Vitamin B_3_ 50 mg.

^2^ Mineral premix provides the following per kg of the die: Fe 60.0 mg, Se 0.3 mg, Mn 80.0 mg, I 0.3 mg, Cu 8.0 mg, Zn 60.0 mg.

^3^ Nutrient levels are calculated values.
